# 

**Published:** 1884-03

**Authors:** 


### CONTENTS:

Art. I.—Recent Notices of Dr. Gorgas' Treatise on "Dental Medicine." By C. L. S...481

Art. II.—Dentistry and Otopathy. By F. Park Lewis, M. D........................486

Art. III.—Etiology of Dental Caries..............................................497

Art. IV.—Treatment of Children's Teeth. By Prof. E. T. Darby..................506

Art. V.—The Causes of the Failure of Gold as a Filling Material.

By Dr. A. A. Blount..........................................514

Art. VI.—Staining Artificial Teeth to Match Exceptional Natural Teeth.

By William Dunn..............................................517

### EDITORIAL, ETC.

Annual Commencement of the Dental Department of the University of Maryland...520
The Maryland Dental Bill.........................................................523

### MONTHLY SUMMARY.

Caries Dentium...................................................................525

Best Method of Producing Anæsthesia.............................................527

